# Current research status on HER2 protein expression levels and the efficacy of targeted therapy in breast cancer

**DOI:** 10.3389/fonc.2025.1551415

**Published:** 2025-06-30

**Authors:** Jiani Liu, Xinyu Peng, Yang Yang, Ruixue Yue, Xuhui Huang, Yongtao Du, Chongzhu Hu

**Affiliations:** ^1^ Department of Breast Surgery, Baoding No.1 Central Hospital, Baoding, Hebei, China; ^2^ Department of Gastrointestinal Surgery, Affiliated Hospital of Hebei University, Baoding, Hebei, China; ^3^ Department of Oncology, Affiliated Hospital of Hebei University, Baoding, China

**Keywords:** HER2 protein expression, breast cancer, targeted therapy, efficacy prediction, treatment strategies

## Abstract

This review summarizes the relationship between HER2 protein expression and the efficacy of three anti-HER2 targeted therapies in breast cancer patients: monoclonal antibodies, antibody-drug conjugates (ADCs), and tyrosine kinase inhibitors (TKIs). The effectiveness of monoclonal antibody therapies positively correlates with HER2 protein expression levels, with HER2 IHC 3+ patients exhibiting better outcomes than IHC 2+/FISH+ patients. In contrast, those with low HER2 protein expression (IHC 1+ or 2+/FISH–) were not beneficial. In patients with HER2-positive breast cancer, the efficacy of T-DM1 is independent of HER2 protein expression levels. Patients with different HER2 expression levels can benefit from T-DXd treatment, with a potential positive correlation with HER2 expression levels. For TKIs, efficacy appeared to be positively correlated with HER2 expression, with HER2 IHC 3+ patients outperforming those with HER2 IHC 1+ or 2+/ISH+. However, high-level evidence to evaluate the relationship between HER2 expression levels and the efficacy of different targeted therapies is lacking. Determining whether HER2 protein expression levels influence treatment outcomes and whether tailored strategies based on HER2 protein expression levels should be implemented holds significant implications for advancing precision medicine in breast cancer.

## Introduction

In 2022, approximately 2.309 million new cases of breast cancer were diagnosed globally, with 667,000 deaths, making it the second most common cancer and the fourth leading cause of cancer-related deaths worldwide (1). In China, 357,000 new female breast cancer cases and 75,000 deaths have been reported, ranking second in incidence and fifth in mortality among female cancers (2). HER2-positive breast cancer accounts for approximately 15–20% of all breast cancers, although this proportion varies across studies (3–7). HER2-positive breast cancer is characterized by aggressive biological behavior and poor prognosis. The advent of anti-HER2 targeted therapies has significantly improved patient outcomes. Currently, three anti-HER2 drugs are clinically available: 1. Monoclonal antibodies, such as trastuzumab, pertuzumab, and inetetamab; 2. Antibody-drug conjugates (ADCs), such as T-DM1 and T-DXd; 3. Tyrosine kinase inhibitors (TKIs) include lapatinib, neratinib, tucatinib, and pyrotinib.

HER2, a transmembrane glycoprotein with a molecular weight of 185 kDa, comprises extracellular, transmembrane, and intracellular domains and serves as the target for anti-HER2 therapies. The first two drug classes target the extracellular ligand-binding domain, whereas the third targets the intracellular tyrosine kinase domain. HER2 protein expression is commonly assessed using immunohistochemistry (IHC), categorized as overexpression (IHC 3+), moderate expression (IHC 2+), or low or ultra-low expression (IHC 1+ or 0) based on the number of stained tumor cells, staining intensity, and completeness of membrane staining.

Despite these advances, some patients fail to benefit from targeted therapy. Predictive biomarkers for therapeutic efficacy, including hormone receptor status, HER2 protein expression levels, HER2 gene copy number, HER2 heterogeneity, histological grade, Ki-67 index, and tumor-infiltrating lymphocytes, have been explored, but no universally recognized predictive factor has been established.

Studies have suggested a correlation between HER2 protein expression and the efficacy of anti-HER2 therapies. However, high-level evidence for this is scarce. This review examines the relationship between HER2 protein expression and the efficacy of various anti-HER2 therapies, providing a foundation for future research.

## Monoclonal antibodies

Trastuzumab was the first drug approved for HER2-positive breast cancer. It binds to the extracellular domain (ECD) IV of HER2, suppressing intracellular HER2 signaling pathways. Pertuzumab binds to ECD II, preventing HER2 heterodimerization with HER1, HER3, and HER4, blocking downstream tumor signaling. Guidelines recommend trastuzumab with or without pertuzumab as the first-line therapy for HER2-positive disease. Studies have indicated that HER2 protein expression levels correlate with the efficacy of trastuzumab with or without pertuzumab. Patients with HER2 IHC 3+ tumors showed better outcomes than those with HER2 IHC 2+/FISH+ tumors, whereas patients with low HER2 expression (IHC 1+ or 2+/FISH–) had no benefit.

Most studies have focused on neoadjuvant therapy. Several investigations have shown that pathologic complete response (pCR) rates are significantly higher in HER2 IHC 3+ patients than in HER2 IHC 2+/FISH+ patients (8–17). Limited data exist on adjuvant and salvage therapies, but the N9831 trial (NCT00898898) suggests that adjuvant trastuzumab improves disease-free survival (DFS) in HER2 IHC 3+ patients, with no benefit for HER2 IHC 0–2+/FISH+ patients (18). Similarly, the CLEOPATRA study (NCT00567190) found a higher proportion of HER2 IHC 3+ patients among long-term responders (19). However, the NSABP B-47 trial (NCT01275677) confirmed that trastuzumab provides no invasive DFS (iDFS) benefit for patients with low HER2 expression(IHC 1+ or 2+/FISH–) (20). Atallah suggests that adjuvant anti-HER2 therapy after surgery can improve breast cancer-specific survival in patients with HER-2 IHC3+ tumors and ER-/HER-2 IHC2+ tumors, but it has no significant impact on patients with ER+/HER-2 IHC2+ tumors (4).

The differential efficacy of monoclonal antibodies may result from: (1) Higher dependence of HER2 IHC 3+ tumors on HER2 signaling, providing more binding sites for antibodies to block signaling and inhibit proliferation (20); (2) Co-expression of estrogen receptor (ER) and HER2 in HER2 IHC 2+/FISH+ tumors, leading to signaling crosstalk and diminished inhibitory effects on proliferation (9).

## Antibody-drug conjugates

### T-DM1

Ado-trastuzumab emtansine (T-DM1) is the first ADC approved for HER2-positive breast cancer. It comprises trastuzumab connected via a stable linker to DM1, a maytansine derivative with a drug-to-antibody ratio (DAR) of ~3.5:1. The KATHERINE trial (NCT01772472) demonstrated that adjuvant T-DM1 therapy significantly improves outcomes compared with trastuzumab in HER2-positive patients with non-pCR following neoadjuvant therapy. Subgroup analysis revealed that both HER2 IHC3+ and HER2 IHC2+/ISH+ patients benefit similarly from T-DM1, with 3-year invasive disease-free survival (iDFS) rates of 85% and 88%, respectively (21). The ATEMPT trial (NCT01853748) showed that in patients with HER2-positive stage I breast cancer, one year of adjuvant T-DM1 significantly improved 3-year iDFS compared to the wTH regimen. The 5-year iDFS rate was 97%, the recurrence-free interval (RFI) rate was 98.3%, the overall survival (OS) rate was 97.8%, and the breast cancer-specific survival (BCSS) rate was 99.4%. Subgroup analysis demonstrated consistent efficacy across all subgroups, regardless of tumor size, hormone receptor status, or HER2 immunohistochemistry (IHC). The 5-year iDFS (96.9% vs. 96.7%) and RFI (98.4% vs. 97.8%) rates were comparable between IHC3+ and IHC ≤2+ patients (22).

These findings suggest that the efficacy of T-DM1 was observed with almost comparable outcomes in patients with HER2 IHC3+ and HER2 IHC2+/ISH+ patients. However, the efficacy of T-DM1 was limited in HER2-low patients.

### T-DXd

Trastuzumab deruxtecan (T-DXd) is a HER2 ADC comprising a humanized HER2 antibody with the same sequence as trastuzumab conjugated to deruxtecan (DXd). Based on the outstanding results from the DESTINY-Breast series (DB studies), T-DXd is considered a groundbreaking ADC drug with significant clinical potential. Key optimizations in its mechanism of action, such as the high cytotoxicity of topoisomerase inhibitors, the use of a tetrapeptide linker technology, a drug-to-antibody ratio of up to 8:1, and enhanced membrane permeability contributing to a powerful bystander effect, are the main reasons behind the impressive efficacy of T-DXd.

The DB-01 study (NCT03248492) demonstrated the efficacy of T-DXd in HER2-positive patients who received multiple lines of prior treatment. In patients who had received a median of six prior lines of therapy, the median progression-free survival (PFS) was 16.4 months, with an overall response rate (ORR) of 60.9%. Notably, HER2 protein expression level did not significantly impact treatment benefit, with no significant difference in ORR between IHC 3+ and IHC 2+/ISH+ patients (63% vs. 46%, P>0.05) (23).

The DB-02 study (NCT03523585) further confirmed that T-DXd significantly outperformed physician’s choice treatment (TPC regimens) in HER2-positive patients after multiple lines of treatment, with a median PFS of 17.8 months vs. 6.9 months and ORR of 69.7% vs. 29.2% (24).

The DB-03 study (NCT03529110) established T-DXd as the standard second-line treatment in HER2-positive metastatic breast cancer, showing superior efficacy to T-DM1. The median PFS was 28.8 months vs. 6.8 months, with a 12-month PFS rate of 75.8% vs. 34.1% (P<0.001), and an ORR of 79.7% vs. 34.2% (25).

The DB-04 study (NCT03734029) demonstrated that, after 1–2 lines of chemotherapy, T-DXd significantly outperformed the TPC regimen in patients with HER2-low (IHC 1-2+/ISH-) advanced breast cancer, with a median PFS of 9.9 months vs. 5.1 months (P<0.001). T-DXd showed excellent efficacy in patients with low HER2 expression (IHC 1+-2+) (26).

At the 2024 ASCO Annual Meeting, the DB-06 study (NCT04494425) results were presented, showing that T-DXd significantly improved median PFS in patients with HER2-low (IHC 1+-2+/ISH-) and HER2-ultralow (0+<IHC<1+) advanced breast cancer compared to TPC, with a median PFS of 13.2 months for both groups. However, no direct comparison has been made between the two groups (27).

The phase 2 DAISY trial demonstrated HER2 expression is a determinant of T-DXd efficacy. The ORR and mPFS are 70.6%(95% CI 58.3–81) and 9.7 months (95% CI 6.8–13) in HER2 IHC 3+ or ISH+ cohort; 37.5%(95% CI 26.4–49.7) and 6.7 months (95% CI 4.4–8.3) in IHC 1+-2+/ISH- cohort; 29.7%(95% CI 15.9–47) and 4.2 months (95% CI 2.0–5.7) in HER2 IHC 0 cohort (28).

These DB series studies and the DAISY trial suggest that patients with varying HER2 protein expression levels can benefit from T-DXd treatment, with the degree of benefit potentially correlated with HER2 expression levels. The median PFS from DB-01, DB-02, and DB-03 studies enrolling patients with HER2-positive breast cancer were 16.4 months, 17.8 months, and 28.8 months, respectively. While the median PFS from DB-04 and DB-06 studies enrolling patients with HER2-low or ultralow breast cancer were 9.9 months and 13.2 months, respectively. IHC 3+ and IHC 2+/ISH+ patients appear to experience greater benefit compared to HER2-low (IHC 1+ or 2+/ISH-) and HER2-ultralow (0+<IHC<1+) patients. This was further confirmed by the DAISY trial. In HER2-positive patients, subgroup analysis of the DB-01 study indicated that HER2 protein expression level (IHC 3+ vs. IHC 2+/ISH+) did not affect the treatment benefit. More prospective studies are needed to compare the efficacy of T-DXd in patients with different HER2 expression levels.

The focality of HER2 expression might attenuate T-DM1 activity, which was unable to induce a bystander effect for surrounding HER2-negative cells due to a non-cleavable linker. This issue was resolved through T-Dxd. A unique feature of T-DXd is its ability to target HER2-low/ultralow patients. This remarkable efficacy appears multifactorial based on enhanced features of T-DXd compared with T-DM1, the ability to deliver a higher dose, and the bystander effect, tackling intratumor HER2 heterogeneity. However, a high percentage of HER2 IHC 0 cells in the tumor and their spatial distribution relative to HER2-overexpressing cells were associated with a decreased response to T-DXd. This may explain the potential correlation between the magnitude of benefit with T-DXd and HER2 expression.

In recent years, several new ADC agents, such as SYD985, RC48-ADC, ZW49and ARX-788, have emerged. However, it remains unclear whether these drugs exhibit differential efficacies in patients with varying HER2 expression levels.

## Tyrosine kinase inhibitors

Currently available tyrosine kinase inhibitors in clinical use include lapatinib, neratinib, tucatinib, and pyrotinib. TKIs are small molecules that target the intracellular catalytic kinase domain of HER2, competing with ATP, blocking phosphorylation and activating downstream signaling cascades. Lapatinib and Tucatinib are reversible, while Neratinib and Pyrotinib are irreversible pan-HER TKIs that target EGFR, HER2, and HER4.

The NALA study (NCT01808573) aimed to compare the efficacy of lapatinib or neratinib in combination with capecitabine in HER2-positive metastatic breast cancer patients, enrolling 621 patients who had received second-line treatment. The study found that the median progression-free survival (PFS) for the neratinib group was 5.6 months, compared to 5.5 months for the lapatinib group (P=0.059) (29). Subgroup analysis revealed that when both groups were combined, the PFS for HER2 IHC3+ patients was superior to that of HER2 IHC2+/ISH+ patients (5.59 months vs 4.17 months, P<0.0001), suggesting that HER2 IHC3+ patients are more sensitive to both lapatinib and neratinib than HER2 IHC2+/ISH+ patients. However, there is no separate analysis of the differences in efficacy between lapatinib and neratinib in HER2 IHC3+ and HER2 IHC2+/ISH+ patients (30).

The Panphila study (NCT03735966) was a single-arm phase II trial that enrolled 69 HER2-positive patients to evaluate the neoadjuvant efficacy of the TCbHPy regimen (docetaxel, carboplatin, trastuzumab, and pyrotinib). Subgroup analysis showed a trend towards higher pathologic complete response (pCR) rates in HER2 IHC3+ patients compared to HER2 IHC2+/ISH+ patients (34/56, 60.7% vs 4/13, 30.8%). However, the sample size of this study was relatively small (31).

The PHILA study (NCT03863223) aimed to compare the efficacy of first-line treatment with docetaxel and trastuzumab in combination with either pyrotinib or placebo in metastatic HER2-positive breast cancer, enrolling 590 patients, including 297 in the pyrotinib group. In the pyrotinib group, the rate of disease progression or death was higher in HER2 IHC1+ or 2+/ISH+ patients (24/61, 39.3%) than in HER2 IHC3+ patients (75/236, 31.8%), although statistical analysis was not performed (32).

Currently, no studies have assessed the relationship between tucatinib efficacy and HER2 protein expression.

Existing studies suggest that the efficacy of tyrosine kinase inhibitors may be related to HER2 protein expression levels, with HER2 IHC3+ patients showing better efficacy compared to HER2 IHC1+ or 2+/ISH+ patients. The differential efficacy may result from: HER2 protein overexpressing tumors showing significantly higher expression of several receptor tyrosine kinases (RTKs), including FGFR4, EGFR, and HER2 itself (33), providing more binding sites for antibodies to block signaling and inhibit proliferation. However, high-level evidence supporting this relationship is still lacking.

## Conclusion and future directions

Currently, there is no universally accepted predictive factor to accurately forecast the efficacy of anti-HER2 targeted therapies. In clinical practice, the relationship between HER2 protein expression and the efficacy of anti-HER2 targeted therapies has gradually gained attention. Existing studies suggest that HER2 protein expression levels are associated with the efficacy of trastuzumab-based therapies with or without pertuzumab-targeted therapies. Patients with HER2 protein overexpression (IHC 3+) exhibit better therapeutic outcomes than those with moderate HER2 protein expression (HER2 IHC2+/FISH+). Conversely, patients with low HER2 protein expression (IHC 1+ or 2+/FISH-) did not benefit from these treatments. The efficacy of T-DM1 was observed with almost comparable outcomes in patients with HER2 IHC3+ and HER2 IHC2+/ISH+ patients. However, the efficacy of T-DM1 was limited in HER2-low patients. All patients, regardless of HER2 protein expression levels, benefited from T-DXd therapy, and the extent of the benefit appeared to be positively correlated with HER2 expression levels. Tyrosine kinase inhibitors (TKIs) such as lapatinib, pyrotinib, and tucatinib may show superior efficacy in HER2 IHC3+ patients compared to those with HER2 IHC2+/ISH+ expression.

This review is more aligned with current clinical practice, and its conclusions hold practical significance for clinical work. The review focuses on analyzing, synthesizing, and summarizing data from multiple rigorous, high-evidence-level large-scale clinical studies, particularly the IHC 3+ and IHC 2+ subgroups (**Table 1**), ultimately concluding that HER2 protein expression level is a robust predictive biomarker for anti-HER2 therapy response. The authors propose that for HER2-dependent breast cancer patients with IHC 3+ status, de-escalation therapy may be considered. One of the most promising advantages of targeted therapy is its ability to minimize chemotherapy-related toxicity while maintaining efficacy, thereby reducing reliance on cytotoxic drugs, improving patients’ quality of life, and lowering healthcare costs. Currently, treatment guidelines do not differentiate between IHC 2+ and IHC 3+ HER2-positive breast cancer patients. However, a shift in therapeutic strategy may be warranted—for instance, combining large and small molecular-targeted agents to enhance efficacy and overcome resistance, or exploring ADCs alone or in combination with TKIs. The ultimate goal is to improve long-term survival and cure rates, which holds profound implications for precision medicine in breast cancer. Challenges in HER2 IHC Interpretation and Emerging Solutions Current HER2 immunohistochemical (IHC) assessment remains hampered by inaccuracy and limitations, including subjective quantification of positive cell percentage, membrane staining intensity evaluation, and exclusion of false positives (e.g., edge artifacts), all of which demand substantial time and expertise. Moreover, temporal and spatial heterogeneity in HER2-positive breast cancer is a key determinant of therapeutic response and resistance. To address these issues, researchers are actively developing novel HER2 detection technologies. For example, optimizing AI models by training them on extensive HER2-stained datasets enriched with metastatic samples could enhance both precision and efficiency, thereby addressing subjectivity and variability concerns. Beyond HER2 status, pathway crosstalk and interactions introduce substantial uncertainty during treatment. Identifying the dominant biological drivers of individual tumors (e.g., whether ER or HER2 signaling primarily governs tumor growth and progression) is critical. Thus, differential gene expression (DGE) analysis of relevant pathways represents a vital direction for future research.

**Table 1:** Comprehensive table of clinical studies on anti-HER2 targeted therapies.

Therapeutic Class	Drug/Regimen	Study Name/ID	Study Phase	Patient Characteristics	Relationship Between HER2 Expression and Efficacy
Monoclonal Antibodies	Trastuzumab ± Pertuzumab	N9831 (NCT00898898)	Adjuvant	HER2-positive breast cancer; trastuzumab adjuvant therapy vs. observation	IHC 3+ patients showed improved DFS, while IHC 0–2+/FISH+ patients derived no benefit.
		CLEOPATRA (NCT00567190)	1st-line metastatic	HER2-positive metastatic breast cancer; trastuzumab+pertuzumab+docetaxel vs. trastuzumab+docetaxel	Higher proportion of IHC 3+ patients among long-term responders.
		NSABP B-47 (NCT01275677)	Adjuvant	HER2-low (IHC 1+ or 2+/FISH–) breast cancer; trastuzumab adjuvant therapy vs. observation	Trastuzumab did not improve iDFS in IHC 1+ or 2+/FISH patients.
Antibody-Drug Conjugates (ADC)	T-DM1	KATHERINE (NCT01772472)	Adjuvant	HER2-positive breast cancer without pCR after neoadjuvant therapy; T-DM1 vs. trastuzumab	3-year iDFS rates were 85% and 88% for IHC 3+ and IHC 2+/ISH+ patients, showing no significant difference.
		ATEMPT (NCT01853748)	Adjuvant	HER2-positive stage I breast cancer; 1-year T-DM1 vs. wTH regimen (trastuzumab + taxane)	5-year iDFS and RFI rates were comparable between IHC 3+ and IHC ≤2+ patients (96.9% vs. 96.7%).
	T-DXd	DB-01 (NCT03248492)	Late-line metastatic	HER2-positive metastatic breast cancer with ≥2 prior lines; T-DXd monotherapy	ORR was 63% in IHC 3+ vs. 46% in IHC 2+/ISH+ patients (P>0.05), with a trend toward better response in high-expression patients.
		DB-02 (NCT03523585)	Late-line metastatic	HER2-positive metastatic breast cancer with ≥2 prior lines; T-DXd vs.tpc	PFS of 17.8 months vs.6.9 months; ORR of 69.7% vs.29.2%
		DB-03 (NCT03529110)	2nd-line metastatic	HER2-positive metastatic breast cancer; T-DXd vs. T-DM1	Direct analysis of HER2 expression was absent, but median PFS was significantly longer with T-DXd (28.8 vs. 6.8 months).
		DB-04 (NCT03734029)	Metastatic	HER2-low (IHC 1+–2+/ISH–) metastatic breast cancer; T-DXd vs. physician’s choice (TPC)	T-DXd significantly improved median PFS (9.9 vs. 5.1 months), benefiting low-expression patients.
		DB-06 (NCT04494425)	Metastatic	HER2-low (IHC 1+–2+/ISH–) and ultra-low (0+<IHC<1+) metastatic breast cancer; T-DXd vs. TPC	Median PFS was 13.2 months in both groups, indicating benefit in low/ultra-low expression patients.
		DAISY (Phase II) (NCT04132960)	Metastatic	Metastatic breast cancer with different HER2 expression (IHC 3+/ISH+, IHC 1+–2+/ISH–, IHC 0); T-DXd monotherapy	IHC 3+/ISH+ group: ORR 70.6%, median PFS 9.7 months;
					IHC 1+–2+/ISH– group: ORR 37.5%, median PFS 6.7 months;
					IHC 0 group: ORR 29.7%, median PFS 4.2 months.
Tyrosine Kinase Inhibitors (TKI)	Lapatinib/Neratinib + Capecitabine	NALA (NCT01808573)	2nd-line metastatic	HER2-positive metastatic breast cancer; neratinib+capecitabine vs. lapatinib+capecitabine	IHC 3+ patients had better PFS (5.59 vs. 4.17 months, P<0.0001) than IHC 2+/ISH+ patients.
	Docetaxel+Carboplatin + Trastuzumab + Pyrotinib	Panphila (NCT03735966)	Neoadjuvant	HER2-positive breast cancer receiving TCbHPy regimen (docetaxel + carboplatin + trastuzumab + pyrotinib)	pCR rate was 60.7% (34/56) in IHC 3+ vs. 30.8% (4/13) in IHC 2+/ISH+ patients.
	Docetaxel+ Trastuzumab + Pyrotinib/Placebo	PHILA (NCT03863223)	1st-line metastatic	HER2-positive metastatic breast cancer; pyrotinib+trastuzumab+docetaxel vs. placebo arm	Higher disease progression/death rate in IHC 1+ or 2+/ISH+ patients (39.3% vs. 31.8%) than in IHC 3+ patients.

However, there is currently a lack of high-level evidence evaluating the relationship between HER2 protein expression levels and the efficacy of different anti-HER2 targeted therapies. High-quality, prospective controlled studies are needed to further clarify the relationship between HER2 protein expression level and the efficacy of various targeted therapies and in the future, treatment strategies can be adjusted individually according to HER2 expression level to improve the precision of breast cancer treatment.

## Data Availability Statement

All data generated or analyzed during this study are available from the corresponding author, Chongzhu Hu, upon reasonable request.
